# A Potential Long-Acting LDL-Cholesterol–Lowering PCSK9 Monoclonal Antibody: Randomized, Placebo-Controlled Phase 1 Studies

**DOI:** 10.1016/j.jacasi.2021.09.002

**Published:** 2021-11-09

**Authors:** Yimin Cui, Xia Zhao, Litong Qi, Xin Li, Zhijun Huang, Guoping Yang, Lei Qian, Huan Deng, Haoyu Li, Yong Huo

**Affiliations:** a Department of Pharmacy, Peking University First Hospital, Beijing, China; b Department of Cardiology, Peking University First Hospital, Beijing, China; c Department of Pharmacy, Third Hospital of Changsha, Changsha, China; d Clinical Trial Research Center, Third Xiangya Hospital of Central South University, Changsha, China; e School of Pharmaceutical Science, Third Xiangya Hospital of Central South University, Changsha, China; f Innovent Biologics, Suzhou, China

**Keywords:** hypercholesterolemia, LDL cholesterol, PCSK9, tafolecimab, AE, adverse event, ALT, alanine transaminase, AST, aspartate transaminase, BMI, body mass index, HDL-C, high-density lipoprotein-cholesterol, IV, intravenously, LDL-C, low-density lipoprotein-cholesterol, PCSK9, proprotein convertase subtilisin/kexin type 9, Q*x*W, every *x* weeks, SC, subcutaneously, TEAE, treatment-emergent adverse effect

## Abstract

The aim of the studies was to evaluate the safety, tolerability, and efficacy of tafolecimab, a novel proprotein convertase subtilisin/kexin type 9 (PCSK9) monoclonal antibody, in Chinese healthy volunteers and patients with hypercholesterolemia. Fifty-eight healthy volunteers (phase 1a) were randomized to receive a single dose of 25, 75, 150, 300, 450, or 600 mg tafolecimab subcutaneously, 75 or 450 mg intravenously, or placebo. Sixty patients with hypercholesterolemia (phase 1b) were randomized to receive 75 or 140 mg tafolecimab every 2 weeks, 300 or 420 mg every 4 weeks, or 450 or 600 mg every 6 weeks subcutaneously or placebo for 12 weeks. Tafolecimab was well tolerated. Adverse events in both studies were either mild or moderate. In the phase 1a study, a single dose of tafolecimab reduced low-density lipoprotein-cholesterol (LDL-C) levels up to 72% in healthy volunteers. In the phase 1b study, tafolecimab reduced LDL-C levels up to 71.6% and by more than 50% consistently to week 12 for all tafolecimab dose regimens. Tafolecimab is a safe PCSK9 monoclonal antibody with significant and potential long-acting LDL-C–lowering effect. (Single Ascending Dose Study of PCSK-9 Inhibitor [IBI306] in Healthy Subjects; NCT03366688) (Multiple Ascending Dose Study of PCSK-9 Inhibitor [IBI306] in Chinese Patients With Hypercholesterolemia; NCT03815812)

Elevated level of low-density lipoprotein-cholesterol (LDL-C) is a major risk factor for cardiovascular diseases. Serum LDL-C and total cholesterol levels have been increasing in the Chinese population in recent years, with hyperlipidemia-induced cardiovascular events estimated to increase by 9.2 million from 2010 to 2030 (1).

Statins are considered the first-line therapy for hyperlipidemia. However, many patients fail to achieve the LDL-C goal even with maximum tolerated statin doses (2). Efforts are needed to identify additional LDL-C–lowering strategies.

In the past decade, proprotein convertase subtilisin/kexin type 9 (PCSK9) emerged as a novel target for the treatment of hypercholesterolemia with the approval of 2 monoclonal antibodies, alirocumab and evolocumab (3). Both were administered every 2-4 weeks and reduced LDL-C levels by approximately 60% (4,5). It would be desirable to develop a PCSK9 inhibitor with durable effect to alleviate injection site reactions and improve long-term medication compliance (6).

Tafolecimab is a fully human IgG2 PCSK9 monoclonal antibody produced through affinity maturation by chain shuffling and complementarity determining region mutagenesis on candidate antibodies discovered from a synthetic human antibody library. The phase 1 studies evaluated the safety, tolerability, and efficacy of tafolecimab in healthy volunteers and patients with hypercholesterolemia.

## Methods

### Ethics

The studies were done in accordance with local laws, the International Conference on Harmonization Good Clinical Practice guidelines, and the ethical principles outlined in the Declaration of Helsinki. Respective ethics committees reviewed the study protocols and approved the studies. These studies were registered with ClinicalTrials.gov (NCT03366688 and NCT03815812).

### Participants and procedures

In the phase 1a study, healthy volunteers aged 18-55 years with LDL-C level 1.8-4.9 mmol/L and body mass index (BMI) 19-28 kg/m^2^ were enrolled. Two participants were assigned to the sentinel cohort to receive 25 mg open-label tafolecimab subcutaneously (SC). The other 56 participants were sequentially assigned to 7 cohorts: 75 mg SC, 150 mg SC, 75 mg intravenously [IV], 300 mg SC, 450 mg SC, 600 mg SC and 450 mg IV. Two participants in the 75 mg SC cohort received open-label tafolecimab, and the remaining 6 participants were randomized 2:1 to receive tafolecimab or placebo. Participants in the remaining cohorts were randomized 3:1 to receive tafolecimab or placebo. Each cohort started dosing after the 14-day safety evaluation of the previous cohort.

The phase 1b study enrolled 60 patients with hypercholesterolemia aged 18-70 years, BMI 18-30 kg/m^2^, fasting LDL-C 2.6-5.7 mmol/L, and fasting triglycerides ≤4.5 mmol/L at screening, and on moderate- to high-dose statins for a minimum of 4 weeks. Patients were enrolled in 6 cohorts: 75 mg every 2 weeks (Q2W), 140 mg Q2W, 300 mg Q4W, 420 mg Q4W, 450 mg Q6W, and 600 mg Q6W. In each cohort, patients were randomized 4:1 to receive tafolecimab or placebo. The treatments lasted for 12 weeks. In addition, the efficacy assessment extended to week 14 in the 600 mg Q6W cohort.

### Outcomes

In both studies, the primary end points were the safety and tolerability of tafolecimab. Data on adverse events (AEs), vital signs, laboratory tests, and electrocardiography were obtained at each study visit throughout the study. AEs were categorized according to the Medical Dictionary for Regulatory Activities system organ classes and preferred terms. The severity of the AEs and the association between an AE and the study drug were assessed by investigators based on prespecified criteria.

Secondary end points included pharmacodynamics, immunogenicity, and efficacy of tafolecimab. Efficacy end points included change from baseline to week 12 in LDL-C, total cholesterol, high-density lipoprotein-cholesterol (HDL-C), and triglycerides levels.

### Statistical analysis

The sample sizes were determined empirically to assess the safety and tolerability of tafolecimab. Participants who received at least 1 dose of tafolecimab or placebo were included in the safety analysis. The efficacy analyses included participants who received tafolecimab or placebo and had at least 1 post-baseline evaluation. For both studies, descriptive statistics were applied for demographic and safety parameters. Student *t* test and Fisher exact test were used for comparison of continuous and categoric efficacy end points, respectively. *P* values were provided with a significant level of 0.05. All statistical analyses were performed with the use of SAS 9.4 (SAS Institute).

## Results

### Participants

In the phase 1a study, 58 healthy volunteers were enrolled, with 44 receiving tafolecimab and 14 receiving placebo. Fifty-six participants completed the study. One participant receiving tafolecimab in the 75 mg IV cohort was lost to follow-up and 1 receiving tafolecimab in the 450 mg IV cohort withdrew consent. In the phase 1b study, 60 patients with hypercholesterolemia were enrolled, with 48 receiving tafolecimab and 12 receiving placebo. Fifty-nine patients completed the study, and 1 receiving tafolecimab in the 450 mg Q6W cohort voluntarily discontinued the study at the safety follow-up period. Demographics and baseline characteristics are summarized in the Table 1.

**Table 1:** Demographics, Baseline Characteristics, and Treatment-Emergent Adverse Events

Demographics and Baseline Characteristics										
**Phase 1a**	**25 mg SC (n = 2)**	**75 mg SC (n = 6)**	**75 mg IV (n = 6)**	**150 mg SC (n = 6)**	**300 mg SC (n = 6)**	**450 mg SC (n = 6)**	**450 mg IV (n = 6)**	**600 mg SC (n = 6)**	**Tafolecimab (n = 44)**	**Placebo (n = 14)**

Age, y	28-30	29.3 ± 6.3	33.3 ± 3.7	28.7 ± 6.5	27.7 ± 5.8	34.3 ± 4.7	29.8 ± 6.8	29.0 ± 6.1	–	29.8 ± 6.0
Male	2 (100)	4 (67)	6 (100)	4 (67)	4 (67)	4 (67)	5 (83)	3 (50)	–	12 (86)
BMI, kg/m^2^	20.7-23.4	22.8 ± 1.4	23.1 ± 1.9	25.2 ± 2.7	23.8 ± 1.8	23.8 ± 2.0	22.1 ± 2.6	23.1 ± 2.4	–	23.8 ± 2.7
LDL-C, mmol/L	2.0-3.0	2.6 ± 0.6	3.2 ± 0.7	3.1 ± 0.8	2.7 ± 0.5	2.8 ± 0.7	2.5 ± 0.3	3.0 ± 0.7	–	3.0 ± 0.6
PCSK9, ng/mL	422.7-588.6	445.3 ± 134.3	500.7 ± 146.5	497.1 ± 70.0	365.7 ± 73.9	429.4 ± 107.1	432.7 ± 55.2	450.4 ± 126.1	–	437.9 ± 82.2

**Phase 1b**			**75 mg Q2W (n = 8)**	**140 mg Q2W (n = 8)**	**300 mg Q4W (n = 8)**	**420 mg Q4W (n = 8)**	**450 mg Q6W (n = 8)**	**600 mg Q6W (n = 8)**	**Tafolecimab (n = 48)**	**Placebo (n = 12)**

Age, y			58.8 ± 6.2	54.5 ± 12.4	53.6 ± 6.4	52.8 ± 13.6	47.1 ± 10.4	55.9 ± 9.8	–	44.3 ± 17.8
Male			3 (38)	1 (13)	0	4 (50)	4 (50)	4 (50)	–	5 (42)
BMI, kg/m^2^			26.2 ± 1.8	24.4 ± 2.2	26.0 ± 2.2	23.0 ± 3.3	24.65 ± 2.1	24.0 ± 1.9	–	24.9 ± 3.3
LDL-C, mmol/L			4.4 ± 0.9	3.8 ± 0.8	3.9 ± 0.4	3.9 ± 0.5	3.6 ± 0.6	3.7 ± 0.6	–	3.8 ± 0.6
PCSK9, ng/mL			546.5 ± 98.0	577.3 ± 130.0	582.6 ± 156.8	532.6 ± 96.4	596.2 ± 131.9	507.7 ± 171.0	–	594.4 ± 134.0

**Treatment-Emergent Adverse Events**										

**Phase 1a**	**25 mg SC (n = 2)**	**75 mg SC (n = 6)**	**75 mg IV (n = 6)**	**150 mg SC (n = 6)**	**300 mg SC (n = 6)**	**450 mg SC (n = 6)**	**450 mg IV (n = 6)**	**600 mg SC (n = 6)**	**Tafolecimab (n = 44)**	**Placebo (n = 14)**

Any event	1 (50)	2 (33)	4 (67)	4 (67)	3 (50)	1 (17)	4 (67)	4 (67)	23 (52)	8 (57)
Adverse events that occurred in ≥5% of participants in phase 1a cohorts (n = 58)										
Upper respiratory tract infection	0	0	2 (33)	2 (33)	0	0	2 (33)	3 (50)	9 (21)	2 (14)
Blood creatine phosphokinase increase	0	1 (17)	2 (33)	1 (17)	1 (17)	0	0	1 (17)	6 (14)	0
Blood uric acid increase	0	0	1 (17)	0	0	1 (17)	0	0	2 (5)	2 (14)

**Phase 1b**			**75 mg Q2W (n = 8)**	**140 mg Q2W (n = 8)**	**300 mg Q4W (n = 8)**	**420 mg Q4W (n = 8)**	**450 mg Q6W (n = 8)**	**600 mg Q6W (n = 8)**	**Tafolecimab (n = 48)**	**Placebo (n = 12)**

Any event			7 (88)	7 (88)	7 (88)	5 (63)	3 (38)	5 (63)	34 (71)	9 (75)
Adverse events that occurred in ≥5% of patients in phase 1b cohorts (n = 60)										
Upper respiratory tract infection			2 (25)	0	3 (38)	3 (38)	0	1 (13)	9 (19)	1 (8)
Blood pressure increase			0	0	2 (25)	0	1 (13)	1 (13)	4 (8)	1 (8)
Asthenia			0	0	4 (50)	0	0	0	4 (8)	0
White blood cell count increase			0	1 (13)	0	0	0	1 (13)	2 (4)	2 (17)
Neutrophil count decrease			0	1 (13)	0	0	1 (13)	0	2 (4)	1 (8)
White blood cells urine positive			0	1 (13)	0	0	2 (25)	0	3 (6)	0
Electrocardiogram QT prolonged			0	0	2 (25)	0	0	1 (13)	3 (6)	0
White blood cell count decrease			0	1 (13)	0	0	1 (13)	0	2 (4)	1 (8)
Arthralgia			2 (25)	0	0	1 (13)	0	0	3 (6)	0

Values are range, mean ± SD, or n (%).

BMI = body mass index; IV = intravenously; LDL-C = low-density lipoprotein cholesterol; PCSK9 = proprotein convertase subtilisin/kexin type 9; Q2W, Q4W, Q6W = every 2, 4, 6 weeks; SC = subcutaneously.

### Safety

Tafolecimab was well tolerated and showed an overall good safety profile in both studies. All treatment-emergent adverse effects (TEAEs) were either mild or moderate in severity, and no serious AE or AE leading to death or treatment discontinuation occurred. In the phase 1a study, TEAEs were reported in 23 participants (52.3%) receiving tafolecimab and 8 (57.1%) receiving placebo. The most common TEAEs were upper respiratory tract infection, blood creatine phosphokinase increase, and blood uric acid increase (Table 1). One participant receiving tafolecimab reported mild alanine transaminase (ALT) and aspartate transaminase (AST) increase. In the placebo group, 1 participant reported mild ALT increase and 1 reported mild blood bilirubin increase.

In the phase 1b study, TEAEs were reported in 34 patients (70.8%) receiving tafolecimab and 9 (75.0%) receiving placebo. The most common TEAEs were upper respiratory tract infection, blood pressure increase, and asthenia (Table 1). One patient receiving tafolecimab reported mild blood bilirubin increase and 1 reported mild ALT and AST increase. In the placebo group, 1 patient reported moderate ALT and AST increase.

Antidrug antibody was detected in 1 participant receiving placebo at baseline in the phase 1a study and in 3 patients receiving tafolecimab during the safety follow-up visit in the phase 1b study. None of the participants developed neutralizing antibody.

### LDL-C and other lipid levels

**Figure 1:**
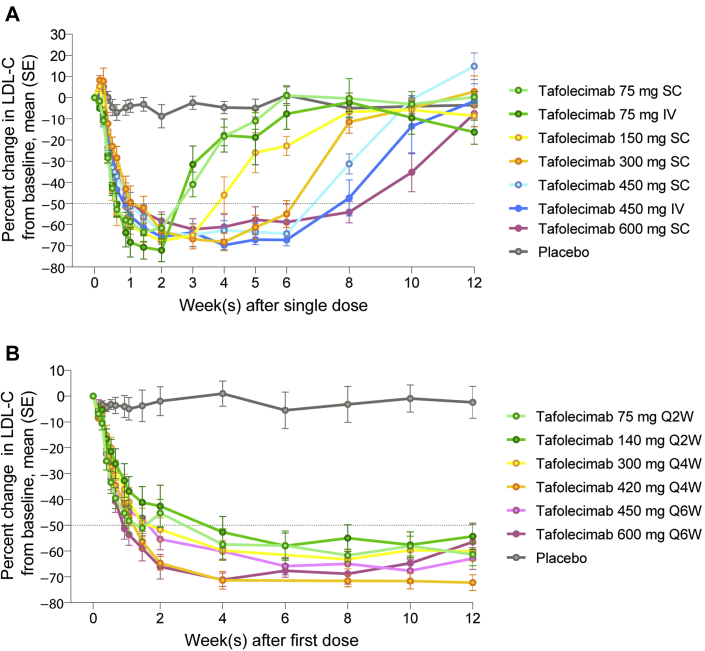
Effects of Tafolecimab on LDL-C Levels Percentage changes from baseline in LDL-C levels in the **(A)** phase 1a and **(B)** phase 1b studies. Error bars indicate standard error (SE). IV = intravenously; LDL-C = low-density lipoprotein cholesterol; Q2W, Q4W, Q6W = every 2, 4, 6 weeks; SC = subcutaneously.

Tafolecimab significantly reduced LDL-C levels in both studies. In the phase 1a study, maximal reduction in LDL-C levels was observed 2 weeks after the single dose. The duration of reductions showed dose dependence. At week 6, LDL-C levels were reduced from baseline by means of 55.0% ± 15.6% (300 mg SC) to 67.2% ± 3.0% (450 mg IV) across the range of tafolecimab doses from 300 mg to 600 mg, whereas the placebo group had an increase from baseline of 1.1% ± 0.9%. Furthermore, 600 mg tafolecimab reduced LDL-C levels from baseline by a mean of 54.1% ± 12.4% at week 8 (Figure 1A).

In the phase 1b study, mean reductions of LDL-C levels by more than 50% were achieved at week 4 and maintained though week 12 with all of the tafolecimab dose regimens (Figure 1B). At week 12, the mean reductions in LDL-C levels ranged from 54.3% ± 14.5% (140 mg) to 72.3% ± 8.5% (420 mg) (*P* < 0.001 for each dose vs placebo, Student *t* test). Moreover, 100% patients receiving tafolecimab (vs 20% receiving placebo) achieved >15% reduction in LDL-C levels (*P* = 0.001, Fisher exact test). Patients in the 600 mg Q6W cohort were monitored up to week 14, when a mean reduction in LDL-C of 43.5% ± 20.9% was achieved in patients receiving tafolecimab, indicating the long-acting LDL-C–lowering effect. In addition, mean reductions in total cholesterol levels from baseline to week 12 ranged from 40.7% ± 13.6% (140 mg) to 56.3% ± 6.3% (420 mg) in patients receiving tafolecimab (vs 0% ± 0.19% in the placebo group). Mean reductions in apolipoprotein B levels from baseline to week 12 ranged from 52.6% ± 19.5% (600 mg) to 68.4 ± 9.5% (420 mg) in patients receiving tafolecimab (vs a 1.2% ± 23.4% increase in the placebo group). Greater improvement in HDL-C, triglycerides, and lipoprotein(a) were observed in patients receiving tafolecimab compared with those receiving placebo.

### PCSK9 levels

In the phase 1a study, maximum reductions in PCSK9 levels, ranging from 89.6% to 100%, were observed 4-24 hours after a single dose of tafolecimab. The duration of reductions exhibited dose dependence. At week 6, the mean reductions in PCSK9 levels in patients receiving tafolecimab in the 450 mg SC and 600 mg SC cohorts were 91.3% ± 9.2% and 89.4% ± 11.3%, respectively. Of note, the mean PCSK9 level in patients receiving tafolecimab in the 600 mg SC cohort was 52.4% ± 76.0% below the baseline at week 8.

In the phase 1b study, 75.6% to 100% reductions in PCSK9 levels were observed across all tafolecimab doses at 24 hours after the first dose. PCSK9 levels remained 50% or more below baseline levels across the range of tafolecimab doses from 140 mg to 600 mg to week 12, when mean reductions in PCSK9 levels of 57.6% ± 53.0% (600 mg) to 93.4% ± 6.2% (420 mg) were achieved.

## Discussion

The studies evaluated the safety and efficacy of tafolecimab in healthy participants and patients with hypercholesterolemia. Although PCSK9 monoclonal antibodies have been approved in the clinic, we are the first to report the clinical investigation of a novel and potential long-acting PCSK9 monoclonal antibody developed in China.

Tafolecimab was well tolerated and showed an overall favorable safety profile, similarly to other PCSK9 antibodies.

In dose-ascending studies of evolocumab, a single dose of 420 mg evolocumab SC reduced mean LDL-C levels up to 67% at day 22, and 420 mg SC Q4W reduced mean LDL-C levels up to 79%. However, in both situations, rapid rebounds were observed after LDL-C levels reached nadirs (7). In our phase 1a study, a 64% mean reduction in LDL-C was achieved and sustained from week 2 to week 6 after a single dose of 450 mg tafolecimab SC. In the phase 1b study, 420 mg tafolecimab Q4W achieved a mean reduction in LDL-C level of more than 70%, which was maintained from week 4 to week 12, 4 weeks after the last dose. Moreover, the Q6W regimens evaluated in our study provided durable reductions in LDL-C levels similar to those of the Q4W and Q2W regimens. Although the magnitude of reductions cannot be compared directly owing to differences in ethnic background and baseline LDL-C levels, the long-acting potential of tafolecimab may offer more convenience to the patients.

## Conclusions

The safety and efficacy demonstrated in these phase 1 studies support further development and investigation of tafolecimab, which is currently being evaluated in Chinese patients with nonfamilial and familial hypercholesterolemia in multiple phase 3 trials.

## Funding Support and Author Disclosures

This study was sponsored by Innovent Biologics and funded by National Science and Technology Major Projects (2019ZX09732-001). Drs Qian, Deng, and H. Li are employees of Innovent Biologics. All other authors have reported that they have no relationships relevant to the contents of this paper to disclose.
